# Management of severe bone loss in patients at risk of medication‐related osteonecrosis of the jaw with microsurgery and guided bone regeneration: A case study

**DOI:** 10.1002/cap.70026

**Published:** 2026-02-19

**Authors:** Pedro Franco Ferreira, José Maurício Paradella de Camargo, Lara Maria Alencar Ramos Innocentini, Ana Carolina Fragoso Motta

**Affiliations:** ^1^ Department of Oral & Maxillofacial Surgery, and Periodontology, Ribeirao Preto School of Dentistry University of São Paulo Ribeirão Preto SP Brazil; ^2^ Instituto Orofacial das Américas Ribeirao Preto SP Brazil; ^3^ Department of Stomatology Public Health and Forensic Dentistry Ribeirao Preto School of Dentistry University of São Paulo Ribeirão Preto SP Brazil

**Keywords:** medication‐related osteonecrosis of the jaw, magnification, microsurgery, guided bone regeneration, case study

## Abstract

**Background:**

Medication‐related osteonecrosis of the jaw (MRONJ) is a challenging complication associated with antiresorptive medications. Its exact pathophysiology remains unclear, but dental extractions and infections are known triggers, complicating prevention, and treatment. While conservative approaches aim to minimize bone exposure, they may be inadequate in severe cases. When surgery is required, results can be unpredictable, potentially complicating rehabilitation and patient satisfaction. This case study explores the potential role of microsurgical techniques and regenerative procedures in treating MRONJ‐risk patients.

**Methods:**

This study presents three cases of female patients over 60 years old, with a history of bisphosphonate use and ongoing denosumab therapy for osteoporosis. Each patient exhibited extensive maxillary bone defects caused by endodontic and/or periodontal disease. Initial conservative treatment was insufficient, due to persistent infection or complications, ultimately requiring surgical intervention. All patients underwent surgery utilizing guided tissue regeneration techniques and biomaterials, following MRONJ‐preventive protocols.

**Results:**

Short‐ and long‐term postoperative follow‐ups, including clinical, radiographic, and tomographic evaluations, demonstrated successful outcome. Patients exhibited improved recovery, bone regeneration, tissue volume preservation, and complete resolution of the infection.

**Conclusions:**

The integration of magnification, microsurgery, and regenerative techniques may provide a viable strategy for managing severe bone loss in MRONJ‐risk patients.

**Key points:**

- Microsurgical techniques and regenerative procedures can be successfully applied in the treatment of severe bone loss in patients at risk of MRONJ.
- When surgery is required in these patients, the approach should focus on minimizing invasiveness while integrating techniques that may enhance oral health and overall quality of life.
- Keys to successful management include accurate diagnosis, careful patient selection, implementation of MRONJ preventive measures, and patient compliance with the postoperative regimen.
- Primary limitations to success involve the unpredictable healing potential of the host and the size and type of the bone defect.

**Plain language summary:**

Some medications used to protect bones in people with osteoporosis can cause rare but serious problems in the jaw, known as medication‐related osteonecrosis of the jaw (MRONJ). This condition often appears after tooth infections or extractions and can make healing very difficult. In this study, we describe three women over the age of 60 years, all receiving osteoporosis medications, who developed large areas of bone damage in the upper jaw due to dental disease. Standard, less invasive care was not enough to control the infection, so surgery was required. To reduce risks, the procedures were performed using a dental operating microscope for precision, along with advanced techniques that encourage bone and tissue regeneration. Special biomaterials were applied to support healing, and all treatments followed careful guidelines designed to minimize the risk of MRONJ. All patients recovered well, with signs of bone regrowth, infection control, and preserved tissue volume both shortly after surgery and during long‐term follow‐up. These cases suggest that combining microsurgery with regenerative methods may offer a safe and effective option for patients at risk of MRONJ, helping them recover more predictably and maintain oral health.

## INTRODUCTION

Medication‐related osteonecrosis of the jaw (MRONJ) is an uncommon but significant complication in dentistry posing considerable challenges in diagnosis and management.^1^, ^2^ It is more frequent in women and is closely associated with the therapeutic use of antiresorptive medications such as bisphosphonates (BPs) and denosumab (DMB). The reported prevalence is less than 5% in patients receiving the antiresorptive therapy for malignancies, and below 0.05% in those being treated for osteoporosis or osteopenia.^2^ However, higher incidences up to 17%, especially with DMB, have been documented in the literature.^3^

Since the initial reports of MRONJ, extensive research has been dedicated to understanding its etiology, pathophysiology, prevention, and treatment.^2^, ^4^, ^5^, ^6^, ^7^ Despite these efforts, the exact mechanisms underlying MRONJ remain unclear.^8^ This uncertainty is particularly concerning given the high prevalence of oral diseases and frequent need of dental extractions, one of the most common triggers for MRONJ.^2^, ^9^, ^10^ These factors further complicate its prevention and management, prompting the use of non‐surgical conservative treatment approaches aimed at minimizing bone exposure.^2^ However, these measures are not always feasible or recommended, especially in cases involving extensive infections affecting the pulp or supporting periodontal structures, where they may difficult proper rehabilitation and fall short of patient expectations.^11^, ^12^

In this context, dentoalveolar techniques, such as guided tissue regeneration (GTR), which involve surgical intervention and therefore carry a potential risk for MRONJ, may nonetheless provide clinical benefits when carefully performed, including improved postoperative recovery, preservation of teeth with severe periodontal compromise and maintenance of tissue volume in extraction sockets.^13^ Furthermore, advances in microsurgery, supported by magnification tools, present an opportunity to make these interventions less invasive, minimizing tissue damage, and preserving structural integrity.^14^, ^15^ This case study aims to describe the potential role of microsurgical techniques and regenerative procedures in the treatment of patients at risk of MRONJ.

## MATERIALS AND METHODS

Three representative cases of extensive bone defect caused by endodontic and/or periodontal disease with a high probability of tissue volume loss were selected from patients with history for BPs and/or DMB seen at a private endodontics and periodontics practice (Paulínia, São Paulo, Brazil) between June 2023 and January 2025. Clinical information and images were retrieved from the electronic health record and the clinical image database. Risk assessments were discussed among the treating dentists, with the patients and with their relatives.

All patients underwent microsurgery using of a dental operating microscope*, following individual medical recommendations and receiving antibiotic prophylaxis. GTR included the application of EDTA 24%† and enamel matrix derivative‡ for root conditioning at the surgical sites, along with heterologous bone graft§ and a collagen membrane.¶ Prescriptions included an antimicrobial mouth rinse (0,12% chlorhexidine/ twice a day for a week), analgesics (dipyrone 1000 mg/ three times a day for 3 days), anti‐inflammatory medication (dexamethasone 4 mg/ twice a day for 2 days—cases 1 and 3—or nimesulid 100 mg/ twice a day for 5 days—case 2). Antibiotics (amoxicillin/clavulanate potassium 875 mg/125 mg) were administered for 07 days, starting the day before the surgery. Additionally, photobiomodulation∥ was applied daily for the first 3 days (808 and 660 nm, 100 mW, 2J per point, 4 points) followed by weekly sessions for 1 month (660 nm, 100 mW, 2J per point, 4 points).

In each case, informed consent was obtained from the patient for publication of the case details. This case study has been reported in line with the PROCESS Guideline ^16^ and received approval from the Institutional Ethics Committees (CAAE: 87475025.2.0000.5419).

## RESULTS

### Case 1

A 72‐year‐old female patient was referred for evaluation of a long‐standing history of periodontal disease, with significant dental calculus accumulation as her only complaint. Her medical history included osteoporosis, managed with oral BPs (alendronate 70 mg weekly for 7 years), followed by DMB (60 mg every 6 months for the past 1.5 years). Additionally, she was undergoing anticoagulant (apixaban) for thromboprophylaxis.

**FIGURE 1:**
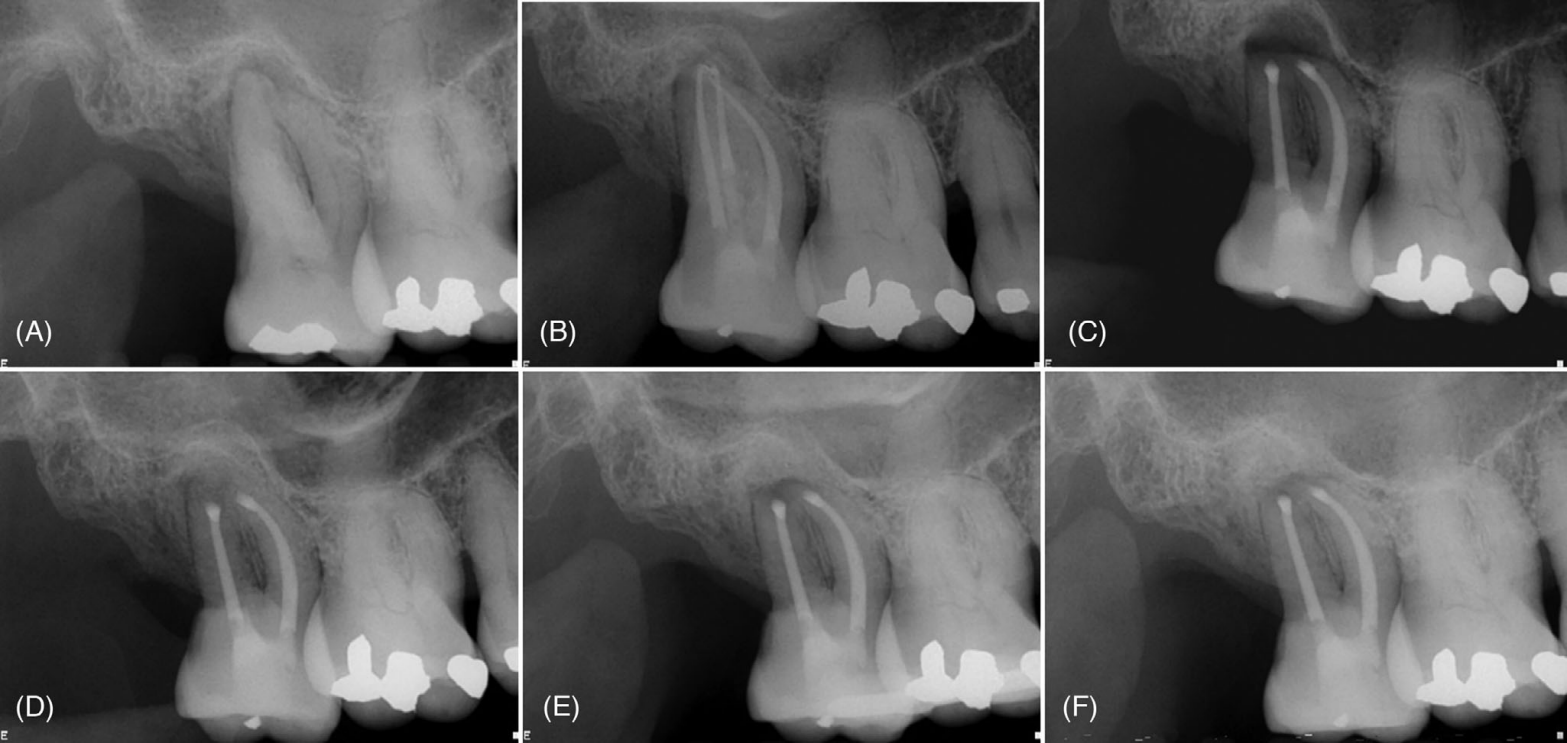
Case 1: Periapical radiographs of tooth #2 showing a radiolucent lesion surrounding the root (A) and post‐root canal treatment (B). Intraoperative radiograph following retropreparation and retrofilling of the buccal canals and complete palatal root resection (C). Immediate postoperative radiograph after guided bone regeneration (D). Radiographs at 1 month (E) and 14 months (F) demonstrating stable healing and bone fill.

**FIGURE 2:**
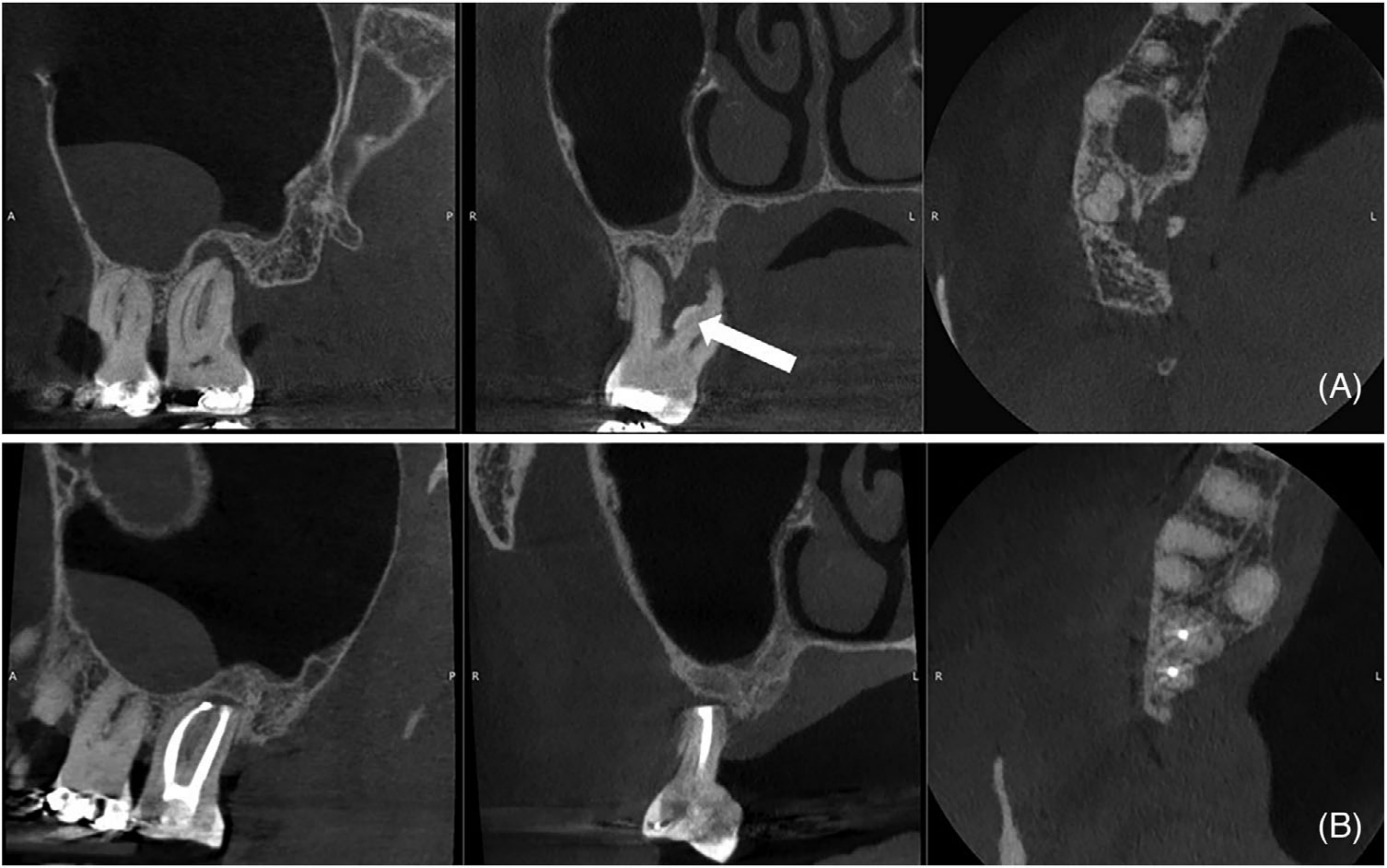
Case 1: Initial CBCT of tooth #2 (sagittal, coronal, axial), showing large hypodense lesion, dental calculus (arrow), furcation caries, and preserved buccal cortical (A). CBCT at 14 months postoperatively (sagittal, coronal, axial), showing bone formation on the distal surface of tooth #2 and apical and palatal remodeling of tooth #1. The hypodense line observed in the apical third of the buccal roots is consistent with repair tissue (collagenization) (B).

Clinical and radiographic examination revealed generalized stage III periodontal disease,^17^ gingival recession, Class III malocclusion, and bilateral posterior crossbite. The patient was asymptomatic, and no tooth loss was observed. Tooth #24 exhibited unsatisfactory endodontic treatment with a periapical lesion, and tooth #2 presented with a periapical radiolucency (Figure 1A), negative pulp vitality test, grade III mobility, suppuration, occlusal trauma, heavy subgingival calculus, and complete exposure of the palatal root. A cone‐beam computed tomography (CBCT) showed an endodontic‐periodontal lesion involving the periapical region of the mesiobuccal (MB) and distobuccal (DB) roots of tooth #2 and confirmed total osseous destruction near the palatal root (Figure 2A). There was a risk of losing the tooth, but considering the history of her antiresorptive therapy, a conservative approach was initially pursued.

Full‐mouth nonsurgical periodontal therapy (NSPT) was performed, followed by regular supportive periodontal care (SPC) every 3 months. Endodontic treatment/retreatment was completed for tooth #2 (Figure 1B) and #24, along with occlusal adjustments. At the 6‐month follow‐up, the patient showed favorable periodontal response with reduced mobility of tooth #2 to grade I. However, biofilm control remained difficult on the exposed palatal root, leading to a recommendation for palatal root resection of tooth #2. Considering the persistence of a periapical radiolucent area and the need for a surgical procedure, complementary surgical endodontic intervention (apicoectomy, retropreparation, and retrofilling) on the buccal roots was also suggested to improve prognosis.

**FIGURE 3:**
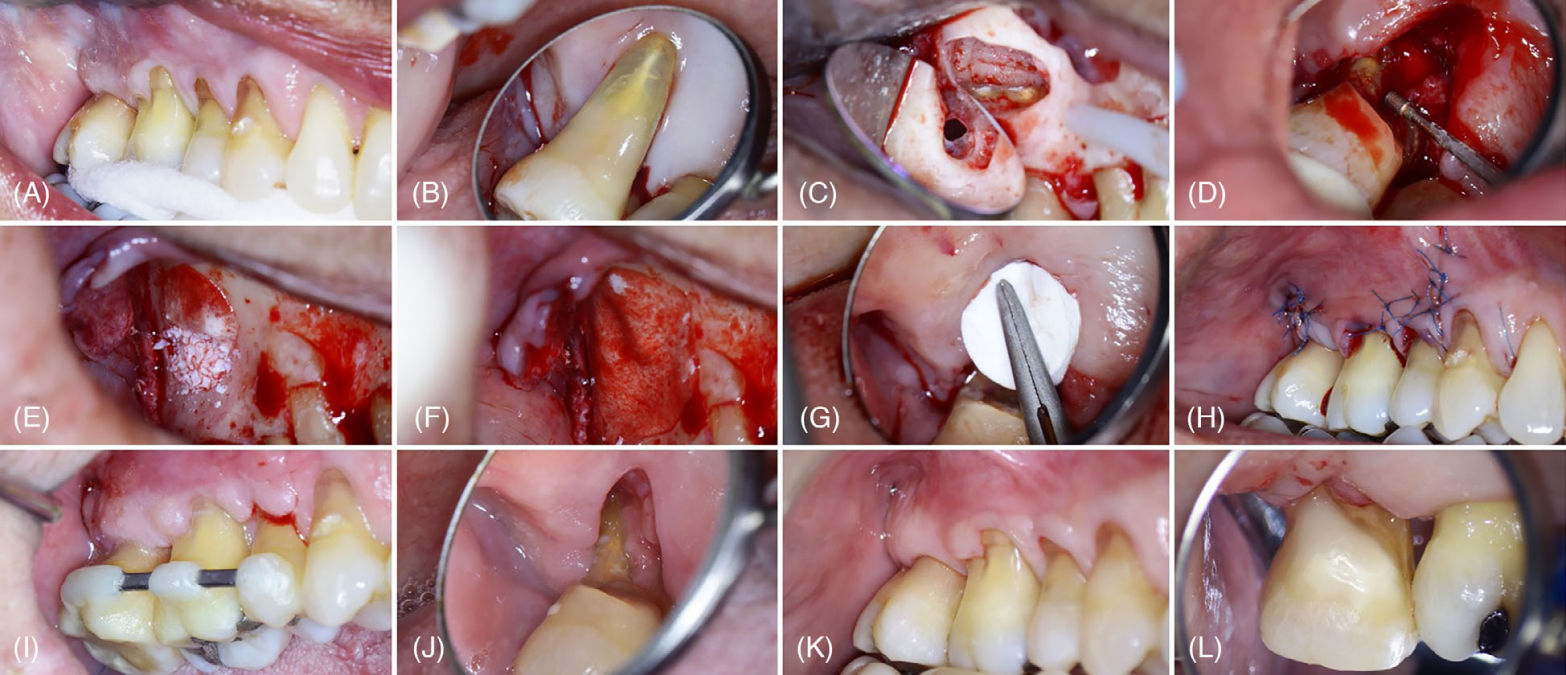
Case 1: Initial buccal view (A). Preoperative palatal view after nonsurgical root scaling, showing exposed palatal root (B). Buccal roots post‐apicoectomy and retrofilling, with sinus membrane perforation (mirror view) (C). Palatal root resection and root/furcation planing using diamond ultrasonic tips (D). Bone graft placement (E). Collagen membrane over graft (F). Placement of a collagen matrix on palatal soft tissue defect (G). Immediate postoperative 6‐0 polypropylene sutures (H). Buccal (I) and palatal (J) view at 2 weeks postoperatively, after teeth splinting and suture removal. Buccal (K) and palatal (L) view at 14 months postoperatively, immediately after restoration for hygiene facilitation.

Medical supervision recommended discontinuing anticoagulant 24 h prior to the procedure, and the patient received antibiotic prophylaxis. During surgery (Figure 3A–H) fenestration of the maxillary sinus was observed, with drainage of serous content. Biomaterials for guided bone regeneration (GBR) were placed without the need for Schneider's membrane suturing (Figure 3E–G). Splinting of the tooth was conducted 2 days postoperatively (Figure 3I). Despite some graft material loss due to accommodation challenges, long‐term follow‐up showed complete tissue repair (Figure 3K,L), resolution of both endodontic and periodontal infection (Figures 1F and 2B), and cessation of tooth mobility.

### Case 2

**FIGURE 4:**
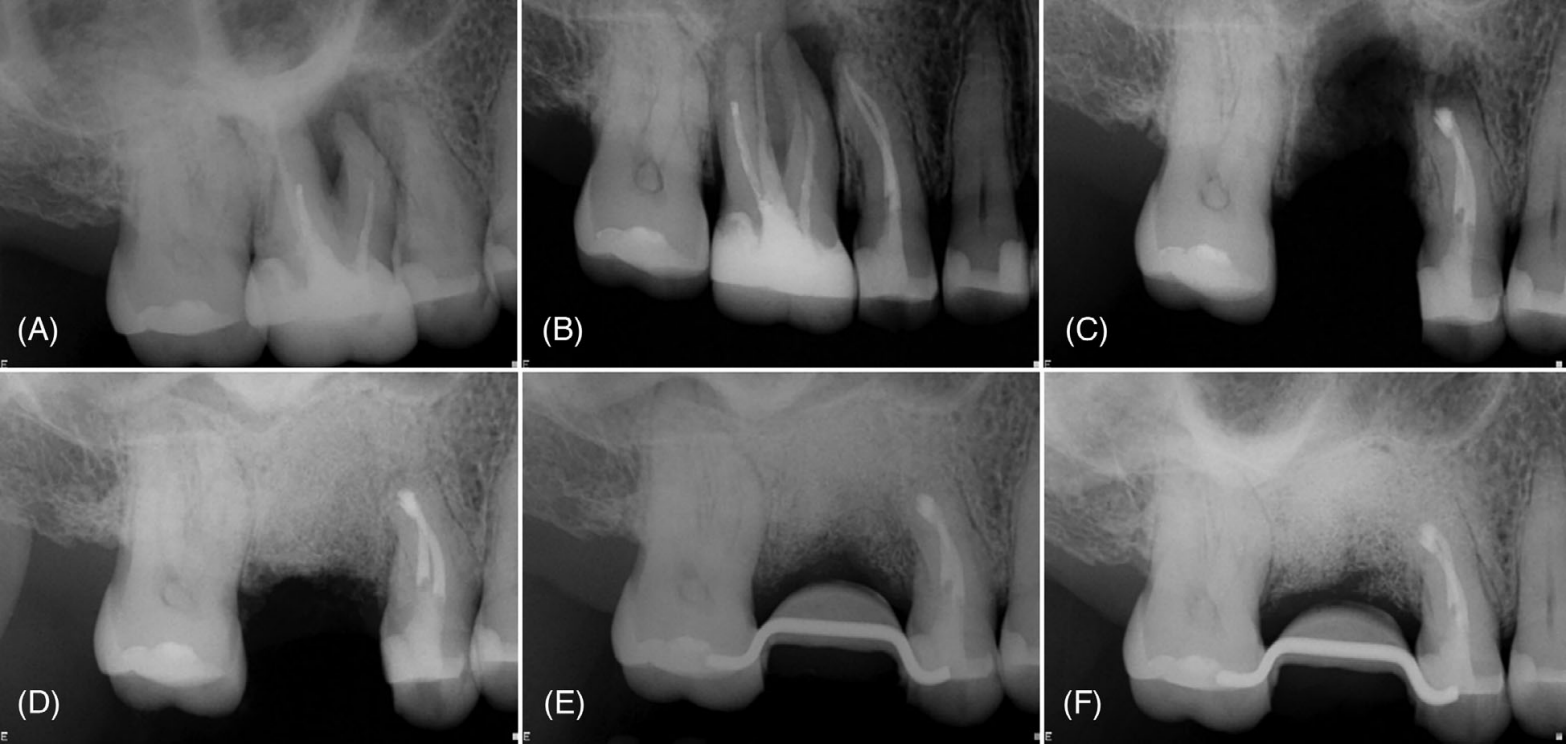
Case 2: Periapical radiograph showing a radiolucent lesion involving teeth #3 and #4 (A). Periapical radiograph after root canal treatment of tooth #4 (B). Intraoperative radiograph following the extraction of tooth #3 and retrofilling of tooth #4 (C). Immediate postoperative radiograph after guided bone regeneration (D). Radiograph at the 1‐month follow‐up, taken after provisional restoration placement for tooth #2 (E). Radiograph at the 8‐month follow‐up (F).

A 63‐year‐old female patient was referred due to an extensive radiolucent lesion around tooth #3, which extended to the adjacent teeth (Figure 4A), detected on routine orthodontic documentation. The patient was asymptomatic, since undergoing endodontic treatment on the tooth #3 over a decade ago. Her medical history included degenerative scoliosisfor which she had undergone multiple spinal fusion surgeries, hypertension, hypercholesterolemia, Parkinson's disease, osteoporosis, gastritis, and depression. The patient was on a polypharmacy regimen, with particular emphasis, for the purpose of this study, on the following medications: acetylsalicylic acid (100 mg—1 tablet/day), calcium malate + vitamin D3 (250 mg + 2.5 mg—1 tablet/day), cholecalciferol (5000 IU—1 tablet/day), and DMB (60 mg—every 6 months, for the past 2 years). She had a history of allergic reactions to ketoprofen, clonazepam, and hydrocortisone.

**FIGURE 5:**
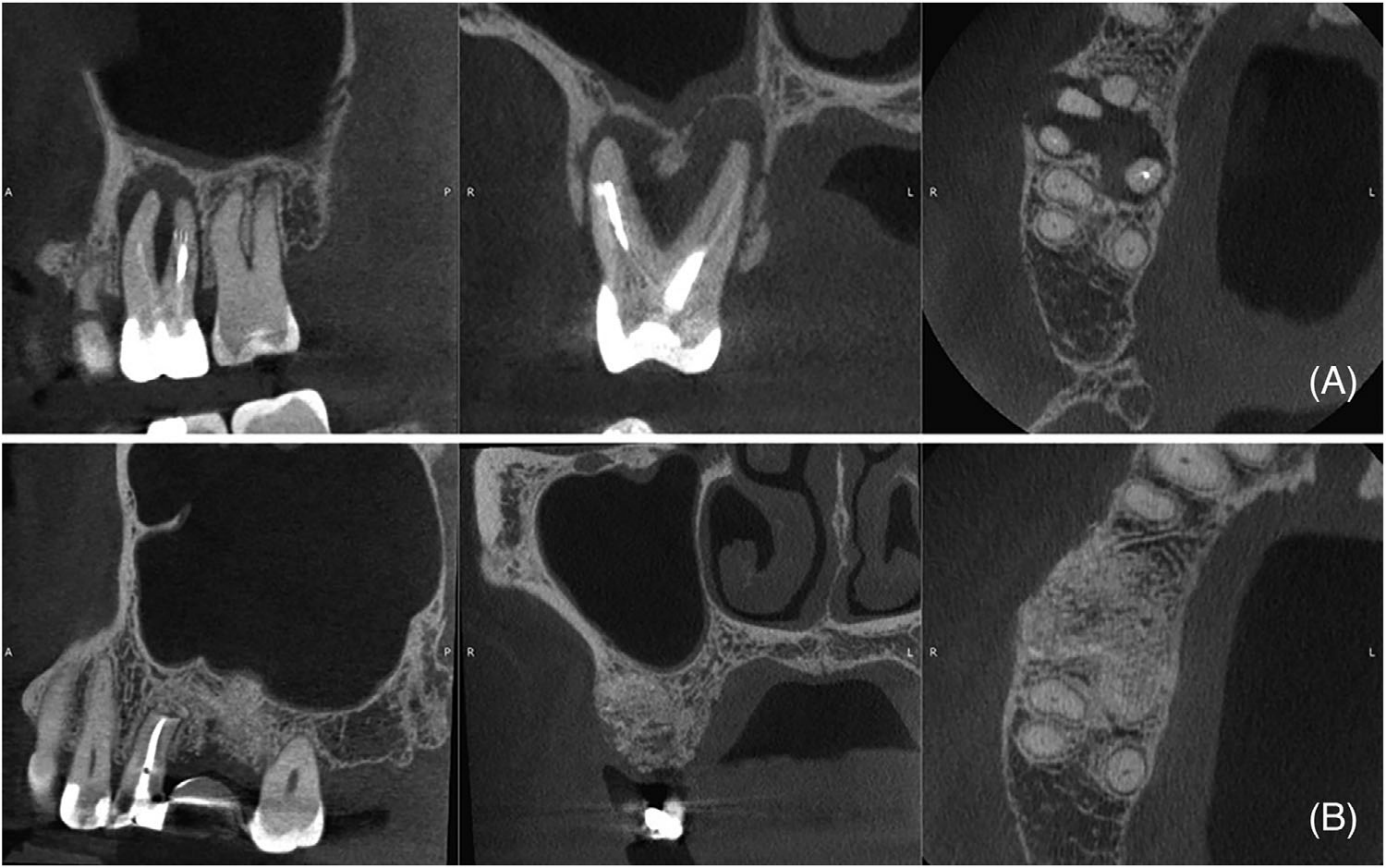
Case 2: Initial CBCT (sagittal, coronal, axial) showing hypodense lesion around teeth #3 and #4, buccal cortical bone fenestration and thickening of the sinus membrane (A). CBCT at 10 months postoperatively (sagittal, coronal, axial), showing bone formation, apical remodeling of tooth #4, preserved tissue volume and normalized sinus membrane (B). CBCT, cone‐beam computed tomography.

**FIGURE 6:**
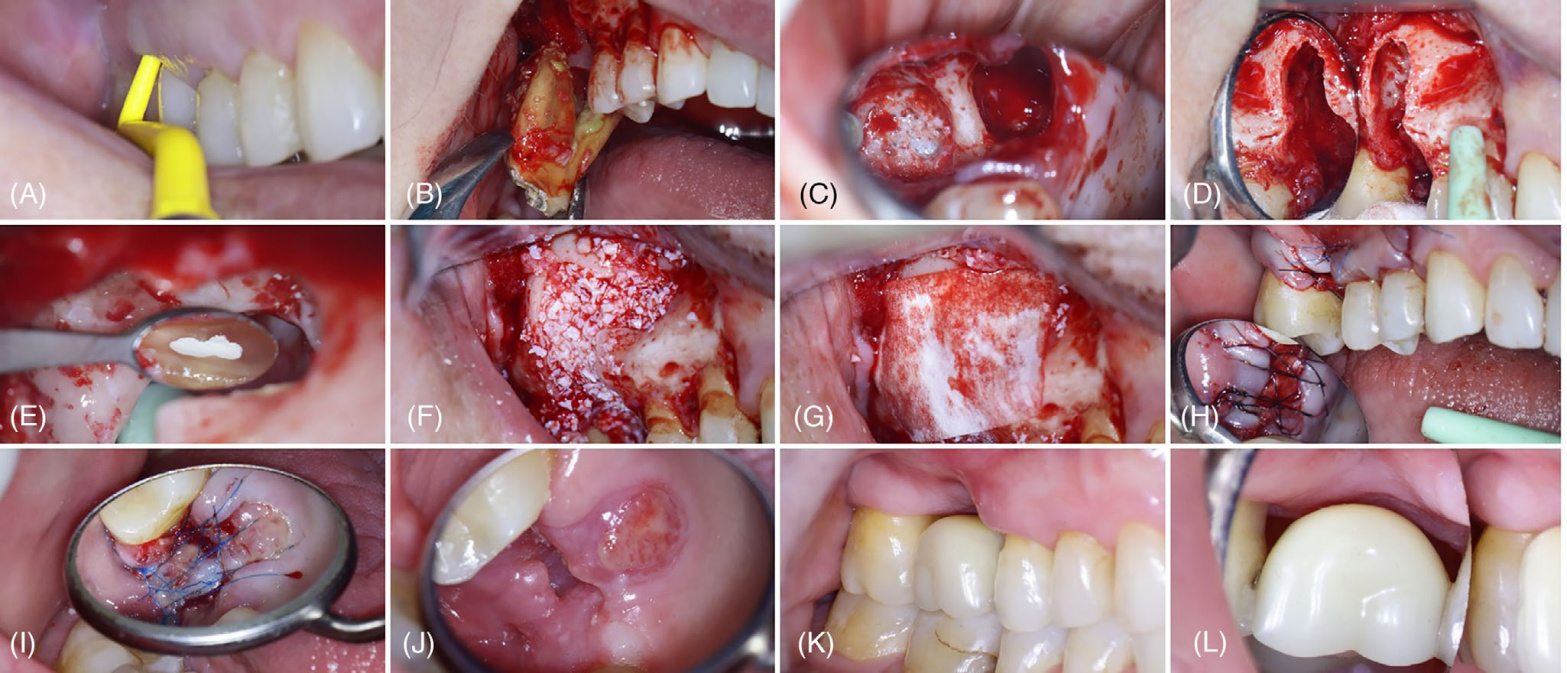
Case 2: Preoperative buccal view of tooth #3 (A). Extraction of tooth #3, showing purulent exudate and granulomatous tissue (B). Alveolus after extraction and curettage, with intact sinus cortical (C). Buccal bone defect (D). Retrofilling of tooth #4 (micromirror view) (E). Heterologous bone graft placement (F). Collagen membrane over the bone graft (G). Immediate postoperative buccal and occlusal (mirror) view with 6‐0 polypropylene and 6‐0 nylon sutures (H). Occlusal view at 1 week (I) and 2 weeks (J) postoperatively. Buccal (K) and palatal (L) views at 8 months postoperatively. A provisional prosthesis was placed 1 month after surgery.

Upon clinical, radiographic, and tomographic evaluation, tooth #3 showed a very large combined endodontic and periodontal lesion, grade 2 mobility, bleeding on probing, and a surrounding probing depth of approximately 15 mm (Figures 5A and 6A). Pulp vitality tests were positive for tooth #2 and negative for teeth #3 and #4. Despite these issues, the patient exhibited generally good oral health, all teeth preserved, with minimal restorative treatment done.

Initial treatment included full‐mouth dental prophylaxis and NSPT performed on upper right quadrant. Endodontic treatment was performed on tooth #4 (Figure 4B) and a microsurgical procedure (Figure 6A–H) was carried out, involving the extraction of tooth #3, SRP for teeth #2 and #4, and apicoectomy/retropreparation/retrofilling for tooth #4. Additionally, alveolar curettage was performed, with no evidence of oroantral communication. Following GTR, the vestibular flap was divided and positioned over the alveolus.

A provisional prosthesis was installed only after its complete closure. The patient showed both radiographic and clinical resolution of the infection (Figures 4E,F, 6K,L). A 10‐month postoperative follow‐up CBCT examination (Figure 5B) confirmed bone volume preservation and complete resolution of the infection. The patient is currently asymptomatic and in the rehabilitation phase of the treatment.

### Case 3

**FIGURE 7:**
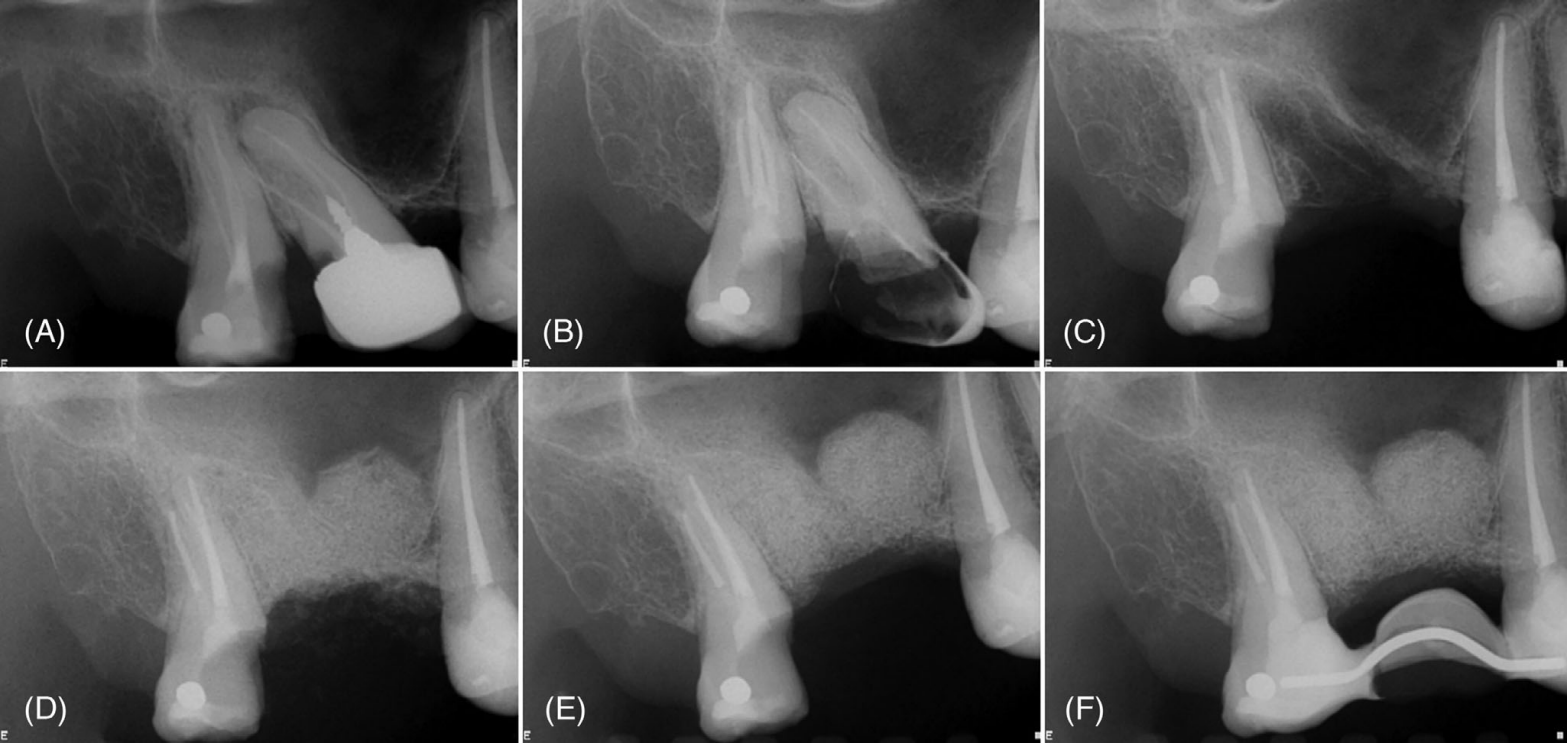
Case 3: Periapical radiograph showing a radiolucent lesion affecting the periapical areas of teeth #1 and #2, interproximal caries, defective restorations, and unsatisfactory root canal treatments (A). Periapical radiograph after root canal retreatment of tooth #1 (B). Intraoperative radiograph following the extraction of tooth #2, alveolar curettage and apicoplasty of tooth #1 (C). Immediate postoperative radiograph after bone regeneration and sinus lift (D). Radiographs at the 1‐month (E) and 3‐month (F) follow‐ups.

A 63‐year‐old female patient was referred to our clinic for a routine examination, reporting no specific complaints. Clinical and radiographic evaluations revealed the need for replacement of several unsatisfactory restorations and prostheses. Additionally, radiolucent areas were detected in the periapical regions of teeth #1, #2, and #14, all of which had undergone inadequate previous endodontic treatments (Figure 7A). The patient's medical history indicated a diagnosis of osteoporosis, for which she had been treated with ibandronate (150 mg once a month for 3 years), followed by risendronate (35 mg weekly for 2 years) and was currently on DMB (60 mg every 6 months).

**FIGURE 8:**
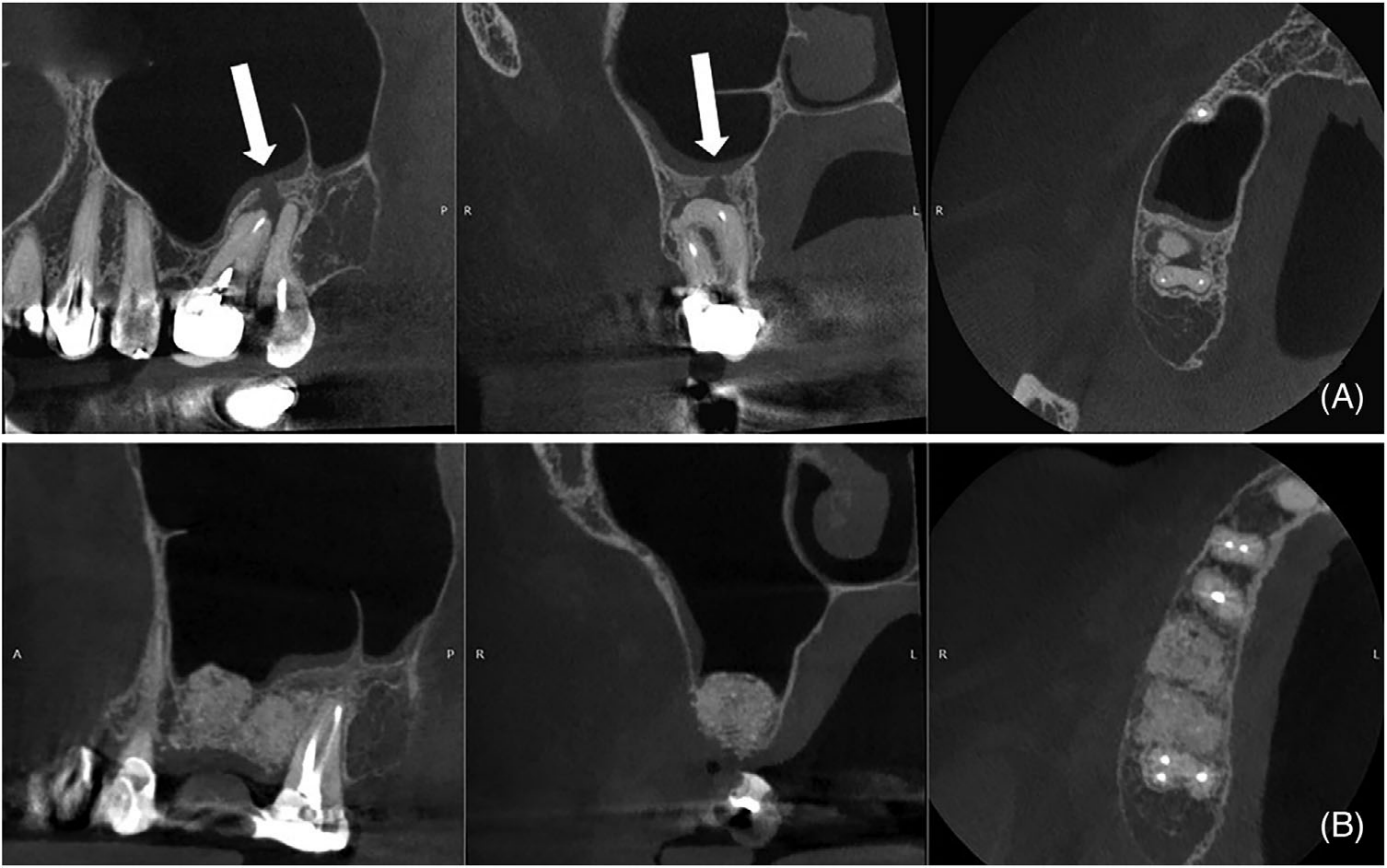
Case 3: Initial CBCT (sagittal, coronal, axial), showing hypodense lesion involving teeth #1 and #2, sinus membrane thickening, cortical fenestration and oroantral communication (arrows) (A). CBCT at 06 months postoperatively (sagittal, coronal, axial), showing bone formation, normalized sinus membrane, cortical repair and closure of oroantral communication (B). CBCT, cone‐beam computed tomography.

A CBCT scan (Figure 8A) was performed to assess the feasibility of endodontic retreatment for teeth #1, #2, and #14, which was deemed viable. Initial steps included comprehensive dental prophylaxis followed by the removal of unsatisfactory prostheses and restorations. Conventional endodontic retreatment was successfully completed for tooth #1 (Figure 7B), as its root structure remained intact. However, despite the attempt at conservative management, after the removal of the unsatisfactory crown, tooth #2 exhibited an extensive vertical root fracture, making retreatment unfeasible.

**FIGURE 9:**
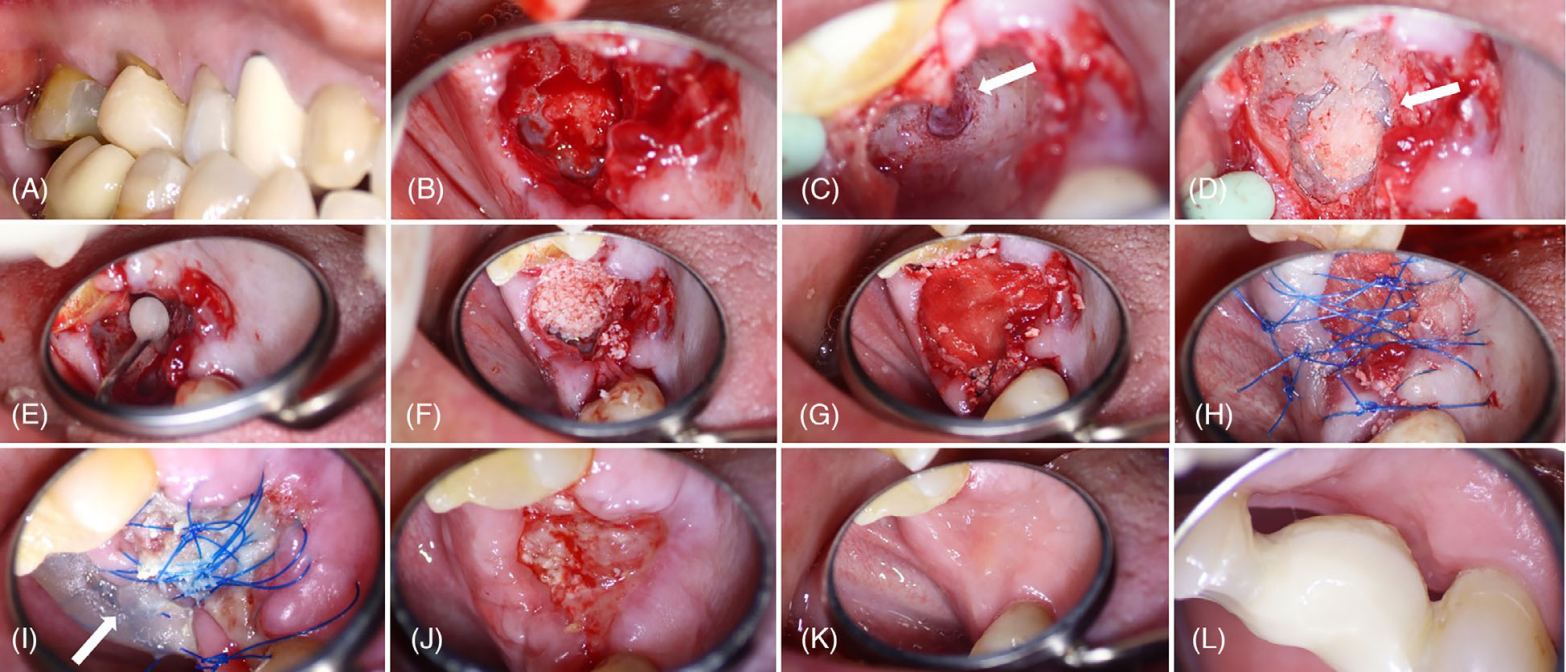
Case 3: Preoperative buccal view of tooth #2 (A). Post‐extraction alveolus before (B) and after (C) the removal of granulomatous tissue, showing the perforated sinus cortical and the intact sinus membrane (arrow). Occlusal view cortical fracture adhered to the sinus membrane (arrow) (D). Enamel‐derived matrix application on the root surface of tooth #1 (mirror view) (E). Heterologous bone graft placement (F). Collagen membrane over the bone graft (G). Immediate postoperative occlusal (mirror) view with 6‐0 polypropylene sutures (H). Occlusal view at 1 week postoperatively showing the placement of a gingival barrier on the sutures (arrow) due to trauma in the buccal mucosa (I). Occlusal view at 2 weeks (J) and 45 days (K) postoperatively. Palatal view at 75 days postoperatively, after provisional prosthesis placement (L).

A microsurgical approach was conducted, which included the extraction of tooth #2, apical curettage of tooth #1 and root planing for teeth #1 and #4 (Figure 9A–H). The alveolar wall fractured during the luxation of tooth #2, but there was no damage to sinus membrane, which reinforced the viability for GBR and sinus lifting through the lateral wall of the alveolus. Although the healing process was slightly prolonged, the patient experienced no pain. After 3 months, complete tissue repair was observed, with preservation of both soft and hard tissue volume which allowed the placement of a provisional prosthesis (Figures 7E,F, 9K,L). The treatment demonstrated complete resolution of the infection and substantial tissue healing (Figure 8B). The patient is currently under observation, with plans for similar procedures in the contralateral arch in the future.

## DISCUSSION

The successful resolution of the presented cases emphasizes the effectiveness of a well‐structured treatment approach for patients at risk of MRONJ. As reported by Ruggiero et al. (2022), pre‐existing inflammatory conditions such as periodontal disease or periapical pathology are recognized risk factors for the development of MRONJ.^2^ Considering the patients’ expectations to eliminate infection while preserving as many teeth as possible, individualized conservative treatment plans were initially formulated, including endodontic treatment and NSPT, followed by SPC. However, due to persistent infection or complications, surgical intervention ultimately became necessary. At this stage, concerns regarding volume loss, along with the patients’ hopes for functional and esthetic improvements, required a careful balance between the risk of the MRONJ development and the benefits of regenerative surgical techniques.

Several key aspects were observed: all patients were female, over the age of 60 years, with a history of BP and DMB therapy for osteoporosis, a condition associated with a lower MRONJ risk compared to malignancies.^2^ Their lesions were located in the maxilla, where MRONJ complications are less frequent than in the mandible.^2^, ^18^ To further mitigate MRONJ risk, all patients who were nearing their next half‐yearly DMB dose were advised, under medical supervision, to postpone it for 3 months, exceeding the minimum 1‐month deferral suggested by Campisi et al.^19^ To optimize surgical outcomes, a comprehensive preoperative protocol was implemented, including meticulous oral hygiene, plaque control and antimicrobial therapy.^2^, ^20^, ^21^ Additionally, photobiomodulation was also employed to promote faster healing and reduce postoperative discomfort.^22^, ^23^

While MRONJ's exact pathophysiology remains unclear, several mechanisms have been proposed, including direct toxicity of BPs to the oral mucosa, altered immune responses, inhibition of angiogenesis and impaired wound healing.^5^, ^24^, ^25^ Woo et al. also attributed the particular vulnerability of the jaws to their higher bone turnover and thin mucosa barrier which normally prevents microbial invasion.^6^ These factors create a biologically unfavorable environment for tissue repair and regeneration, making surgery and regenerative techniques generally contraindicated in MRONJ‐risk patients.^13^ However, literature suggests that when MRONJ is already established, surgical intervention leads to better outcomes.^26^, ^27^, ^28^, ^29^, ^30^ Reported approaches include local debridement, osteoplasty, segmental osteotomy, and free vascularized tissue reconstruction, as non‐surgical treatments often fail to achieve complete lesion healing.^29^, ^31^

In this context, minimally invasive surgical techniques and regenerative procedures have been explored as strategies to address periodontal and endodontic bone defects, making these interventions both more effective and less traumatic.^32^, ^33^, ^34^ By improving visibility, the use of magnification tools, such as surgical loupes and dental operating microscopes, allows for finer tissue manipulation, faster recovery times and a lower likelihood of complications.^14^, ^15^ Such approaches may offer particular advantages for patients at risk of MRONJ, as they align with the principles of reducing surgical trauma and promoting favorable healing conditions. Additionally, promising results have been observed in MRONJ‐risk patients undergoing procedures such as vertical ridge augmentation using bone grafts and a titanium mesh, alloplastic graft placement, maxillary bone ridge splitting, implant placement, mesenquimal cells with heterologous bone placement, and platelet‐rich fibrin (PRF) applications for wound healing and tissue regeneration.^13^, ^35^, ^36^, ^37^ One animal study further demonstrated that grafts improved bone remodeling even in the presence of antiresorptive medications.^38^

Beyond surgical considerations, managing patients at risk of MRONJ also involves addressing their perceptions about the antiresorptive therapy. In some cases, exaggerated concerns about MRONJ development may lead patients to discontinue or refuse treatment, potentially compromising the therapeutic benefits of these medications.^2^, ^4^, ^39^ Effective care should not only focus on preventing MRONJ complication but also on addressing patients’ long‐term rehabilitation needs and incorporating their preferences into treatment decisions.^40^ Patients in this category are frequently advised to undergo extraction as a definitive solution, which may represent overtreatment, particularly when conservative or minimally invasive alternatives are feasible. When surgery is already required, efforts should be made to minimize invasiveness while integrating techniques that support optimal oral health and overall quality of life.

## CONCLUSIONS

In conclusion, priority was given to an approach combining minimally invasive periodontal and endodontic techniques, with an emphasis on careful tissue handling. Although this case study does not establish a treatment guideline, it illustrates that the integration of magnification, microsurgery, and regenerative techniques combined with close postoperative follow‐up can be a viable and effective option for managing patients at risk of MRONJ, achieving predictable short‐ and long‐term outcomes.

## AUTHOR CONTRIBUTIONS

P.F.F. drafted the manuscript, conceived the study, performed all procedures under the dental operating microscope, and was responsible for the clinical images and follow‐up. J.M.P.C. and L.M.A.R.I. contributed to treatment planning and revised the manuscript. A.C.F.M. conceived the study and revised the manuscript. All authors have read and agreed to the published version of the manuscript.

## CONFLICT OF INTEREST STATEMENT

The authors declare no conflicts of interest.

## Footnotes

* OPMI MD, Zeiss, Oberkochen, Germany.

† PrefGel, Straumann, Basel, Switzerland.

‡ Emdogain, Straumann, Basel, Switzerland.

§ Bio‐Oss Small, Geistlich, Wolhusen, Switzerland.

¶ Bio‐Gide, Geistlich, Wolhusen, Switzerland.

∥ Therapy XT, DMC, São Carlos, Brazil.
